# The Impact of Weighting Factors on Dual-Energy Computed Tomography Image Quality in Non-Contrast Head Examinations: Phantom and Patient Study

**DOI:** 10.3390/diagnostics15020180

**Published:** 2025-01-14

**Authors:** Doris Šegota Ritoša, Doris Dodig, Slavica Kovačić, Nina Bartolović, Ivan Brumini, Petra Valković Zujić, Slaven Jurković, Damir Miletić

**Affiliations:** 1 Department of Medical Physics and Radiation Protection, Clinical Hospital Center Rijeka, Krešimirova 42, 51000 Rijeka, Croatia; segota.doris@gmail.com; 2 Department for Medical Physics and Biophysics, Faculty of Medicine Rijeka, Braće Branchetta 20, 51000 Rijeka, Croatia; 3 European Telemedicine Clinic S.L., C/Marina 16-18, 08005 Barcelona, Spain; 4 Department of Diagnostic and Interventional Radiology, Clinical Hospital Center Rijeka, Krešimirova 42, 51000 Rijeka, Croatia; 5 Department of Radiology, Faculty of Medicine, University of Rijeka, Braće Branchetta 20, 51000 Rijeka, Croatia; 6 Department of Anatomy, Faculty of Medicine, University of Rijeka, Braće Branchetta 20, 51000 Rijeka, Croatia; 7 Department of Radiological Technology, Faculty of Health Studies, University of Rijeka, Ul. Viktora Cara Emina 5, 51000 Rijeka, Croatia

**Keywords:** anthropomorphic head phantom, contrast-to-noise ratio, dual energy head CT, protocol optimization, weighted average images, weighting factors

## Abstract

**Background**: This study aims to evaluate the impact of various weighting factors (WFs) on the quality of weighted average (WA) dual-energy computed tomography (DECT) non-contrast brain images and to determine the optimal WF value. Because they simulate standard CT images, 0.4-WA reconstructions are routinely used. **Methods**: In the initial phase of the research, quantitative and qualitative analyses of WA DECT images of an anthropomorphic head phantom, utilizing WFs ranging from 0 to 1 in 0.1 increments, were conducted. Based on the phantom study findings, WFs of 0.4, 0.6, and 0.8 were chosen for patient analyses, which were identically carried out on 85 patients who underwent non-contrast head DECT. Three radiologists performed subjective phantom and patient analyses. **Results**: Quantitative phantom image analysis revealed the best gray-to-white matter contrast-to-noise ratio (CNR) at the highest WFs and minimal noise artifacts at the lowest WF values. However, the WA reconstructions were deemed non-diagnostic by all three readers. Two readers found 0.6-WA patient reconstructions significantly superior to 0.4-WA images (*p* < 0.001), while reader 1 found them to be equally good (*p* = 0.871). All readers agreed that 0.8-WA images exhibited the lowest image quality. **Conclusions**: In conclusion, 0.6-WA reconstructions demonstrated superior image quality over 0.4-WA and are recommended for routine non-contrast brain DECT.

## 1. Introduction

Over the last decade, dual-energy computed tomography (DECT) has been integrated into routine clinical practice [1] and has since found numerous clinical applications [2]. DECT is an imaging method that simultaneously utilizes two X-ray beams of different energies. These beams are generated by voltages of 70–80 kVp and 140–150 kVp. Low-energy X-ray beams yield larger contrast between different tissues due to the dominant interaction mechanism being the photoelectric effect. Conversely, high-energy X-ray beams result in lower noise and fewer artifacts because the dominant interaction mechanism is Compton scattering [3].

In standard single-energy CT (SECT) brain imaging, a single kilovoltage value is selected to generate the X-ray beam, usually 100 kV or 120 kV. This provides only a compromise between the achievable contrast-to-noise ratio (CNR) of the brain tissue at acceptable artifact levels in image data [4]. Generally, the higher the contrast between normal tissue, the greater the ability to identify pathology. The capacity to increase the CNR is pivotal for improving the diagnostic accuracy of brain CT, as the inherent difference in attenuation between the gray matter (GM) and white matter (WM) is only 5–10 HU [5]. Additionally, the quality of SECT images of the brain is frequently reduced by beam hardening artifacts caused by X-ray beam passage through thick bone or hardware [6].

The primary advantage of DECT over SECT is numerous post-acquisition data processing capabilities without additional patient irradiation. Some of the most important include the generation of virtual monoenergetic reconstructions, weighted average (WA) fused reconstructions, tissue/material characterization and decomposition, and the generation of iodine maps [7,8]. Additionally, many studies have shown that DECT improves artifact reduction from metal components [9,10] and has also been utilized in radiotherapy treatment planning, as reported by some authors [11,12]. Some of the most useful DECT features in neuroradiology include the DECT-based bone removal technique in intracranial CT angiography, which is effective in elucidating cerebral vascular anatomy [13,14,15], artifact reduction from metal components [16,17], differentiation of similarly appearing hyperdense materials such as iodine, calcification, and blood, differentiation of contrast extravasation from acute bleeding after interventional stroke treatment [18,19], and detection of acute stroke on virtual monoenergetic reconstructions and iodine maps, which have been proven superior to SECT [20,21].

Several approaches in acquiring DECT are available from different vendors, including sequential acquisition of scans with different energies, rapid kV switching, multilayer detectors, and dual-source CT scanners [22]. A dual-source DECT scanner (Siemens Definition Flash, Siemens, Forchheim, Germany) is installed in our hospital. Dual-source CT enables the simultaneous acquisition of image data at different tube voltages, with one tube typically operating at 80 kVp and the other at 140 kVp. Scanning the head at these two different energies enables reconstructions with different mixed energy ratios or virtual monochromatic reconstructions, which enables optimization of different aspects of image quality. Reconstructions with a lower mixed energy ratio or higher keV reduce streak artifacts through the skull base, which often limit the evaluation of posterior fossa structures. Reconstructions with a higher mixed energy ratio or lower keV improve the contrast between GM and WM. Although DECT offers numerous post-acquisition possibilities, having additional image datasets for each patient prolongs radiologists’ evaluation time, affects the workflow, and requires a dedicated DE software-equipped workstation (syngo.via, version VB60A, Siemens Healthineers) [23]. Therefore, WA reconstructions, derived by blending data from high and low energies at a ratio that best resembles SECT images at 120 kV, are automatically generated and used for routine evaluation of DECT brain studies. WA reconstructions at a weighing factor (WF) of 0.3 or 0.4 are accepted as an SECT proxy [24,25,26]. This means that the WA images are reconstructed by using 30% or 40% of low-energy data and 70% or 60% of high-energy data, respectively.

Since the chosen SECT kV does not represent a desirable energy value for brain scanning, it seems counterintuitive to use WA DECT reconstructions that would mimic these SECT images, despite the possibility of optimizing WA images by adjusting the WF. Changing the WF affects image quality metrics, especially image contrast and noise: images at higher WF have the benefit of better contrast but with increased noise, while with a lower WF, noise and artifacts are reduced at the expense of a lower CNR ratio [24,27]. Therefore, it is essential to fully exploit the possibilities of WA reconstructions by investigating the diagnostic potential of different WF values to find the optimal balance between CNR and artifacts for routine evaluation of brain DECT.

Most studies on non-contrast head DECT have primarily focused on comparing image quality and radiation dose with SECT, consistently demonstrating the superiority of DECT [28,29,30]. Only a few studies have investigated the application of X-map algorithms [31] and bone removal efficiency [32] on image quality in non-contrast head CT. To the best of our knowledge, no evaluation of different WA reconstructions on the quality of non-contrast head CT has been performed. Similar studies have only been performed on post-contrast head and neck examinations and post-contrast abdominal and angiographic imaging. In post-contrast abdominal and angiographic imaging, better diagnostic quality of image data was achieved with a WF of 0.5 compared to a WF of 0.3 [25,33], while in post-contrast DECT of the neck, a WF of 0.6 was shown to be superior for lesion detection compared to the recommended SECT-equivalent images acquired with a WF of 0.3 [23]. Paul et al. showed that GM–WM CNR in the temporal lobe of the post-contrast DECT of the brain is highest for 0.6-WA reconstructions [34]. Although all these studies suggest that other WFs should be used instead of those recommended as an SECT proxy, they have investigated the diagnostic quality of post-contrast scans that are different to routine non-contrast head CT. Therefore, we are confident that evaluating the image quality of non-contrast WA reconstructions at different WFs could improve the diagnostic accuracy of routine DECT head examinations.

This study aims to address the underutilization of the full potential of WA images in DECT of the brain. In particular, it evaluates the impact of various WFs on the image quality of non-contrast DECT head examinations. Both phantom and patient studies are used to determine the optimal WF for routine clinical use. The aim is to achieve optimal overall image quality of WA reconstructions by balancing GM–WM contrast and noise-induced artifacts.

## 2. Materials and Methods

### 2.1. Phantom Study

**Figure 1:**
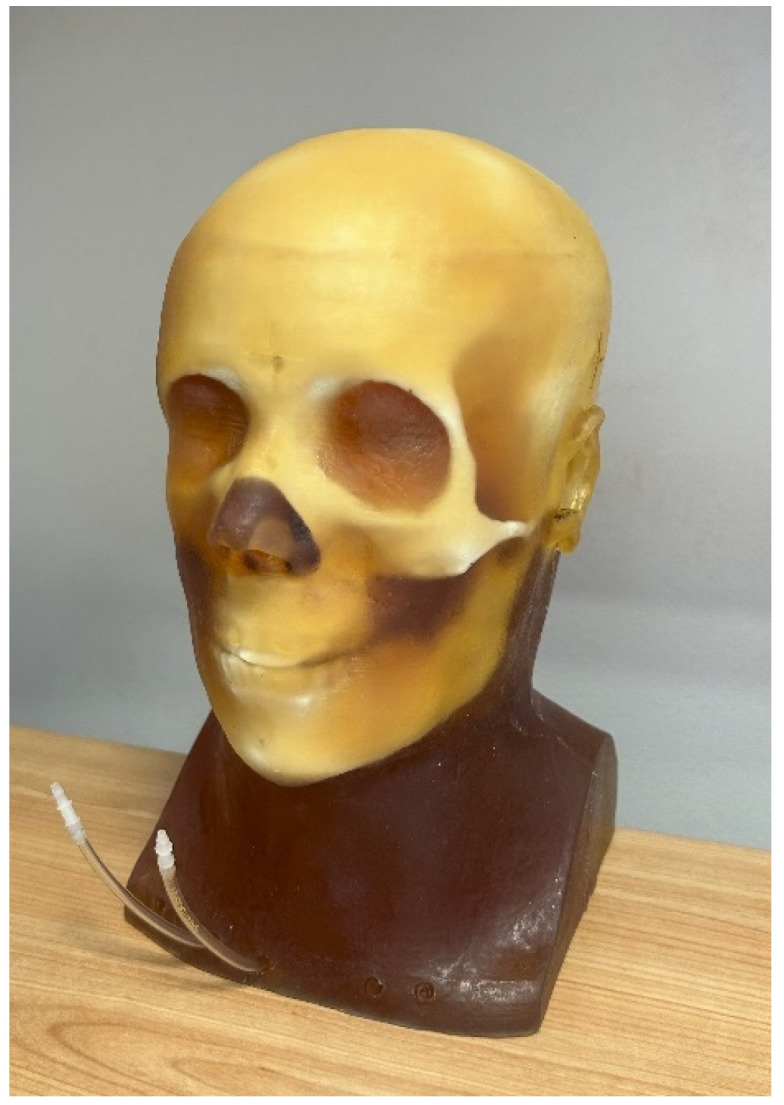
True Phantom anthropomorphic head phantom.

An anthropomorphic head phantom simulating intricate anatomy of the human head (True Phantom Solutions, Windsor, ON, Canada), was used (Figure 1). The phantom was customized based on user requirements and consists of the following parts: anatomically accurate skull bones, brain parenchyma with materials that mimic gray and white matter, features of blood vessels, brain ventricles, and a realistic skin section with X-ray absorption and scattering properties similar to human tissue.

#### 2.1.1. CT Protocol and Image Processing

**Figure 2:**
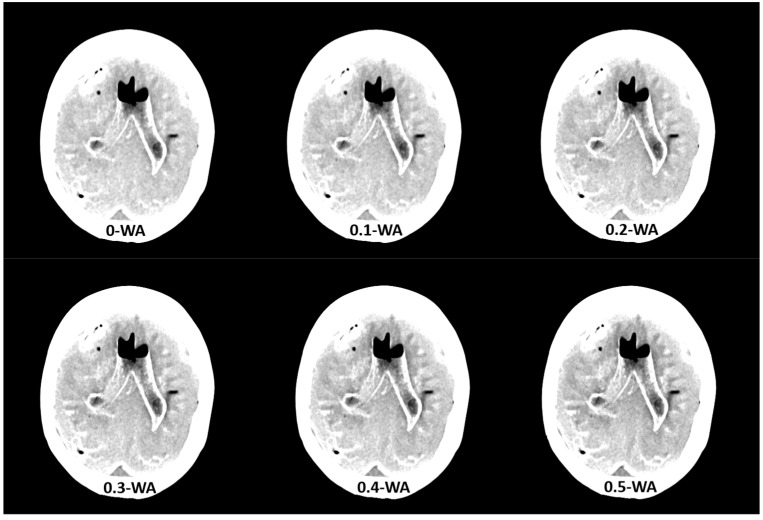

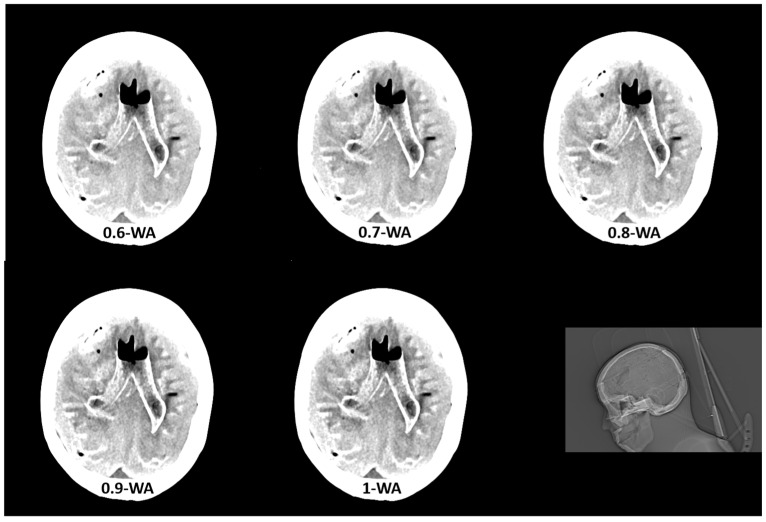
Weighted average (WA) image datasets with different weighting factors (0 to 1) on an anthropomorphic phantom at the level of basal ganglia with the scout image. All images are displayed using the same window width and window level (WW 80, WL 35).

The phantom was scanned with the standard DECT non-contrast head protocol which is used in our hospital on the dual source Siemens Definition Flash scanner. Acquisition is performed at 80 kVp and Sn 140 kVp, where Sn indicates tin filtration of the high- energy tube, with automatic tube current modulation (Care Dose 4d), a pitch of 0.7, and a rotation time of 0.5 s, with XCare on. Quality reference mAs values were 310 for tube A at 80 kV and 155 for tube B at Sn140 kV. The detector configuration was 40 × 0.6 mm. WA reconstructions were generated with WFs from 0 to 1, in 0.1 increments (Figure 2), using the Siemens iterative reconstruction algorithm (SAFIRE) at level 3. The axial soft tissue and bone reconstructions were sent to the Picture Archiving and Communication System (PACS) and to a dedicated workstation.

#### 2.1.2. Quantitative Image Analysis

**Figure 3:**
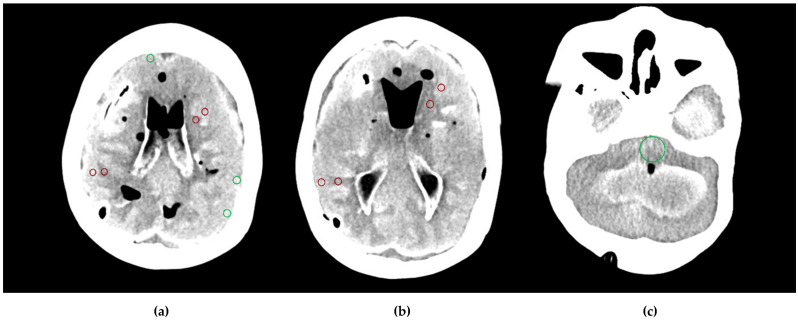
Region of interest (ROI) placement on the cross-sectional phantom images: dark red ROIs represent Hounsfield units (HU) and standard deviation (SD) measurements in gray matter–white matter pairs in the frontal and parietal lobes at the level of the basal ganglia (**a**,**b**), and green ROIs represent SD measurements in the subcalvarial region and the posterior fossa (**a**,**c**).

A methodology similar to that described by Weinman [29], Pomerantz [35], and Kim [25] was applied for quantitative image quality analysis. A fixed circular 25 mm^2^ region of interest (ROI) was placed at four pairs of identical GM and WM locations in the frontal and parietal lobes at the level of the basal ganglia (Figure 3), where the mean and standard deviation (SD) of the attenuation in Hounsfield units (HU) were measured. Posterior fossa and subcalvarial beam hardening artifacts were quantified as the mean SD of three measurements within fixed circular 200 mm^2^ and 25 mm^2^ ROIs, placed at the level of the midbrain and in the brain parenchyma near the skull, respectively (Figure 3). Noise values (SD) for SCA and PFAI were averaged over three measurements to ensure more precise assessment. The CNR of each GM–WM pair was calculated using the following expression:

$$CNR=\frac{mean\;HU\;of\;GM-mean\;HU\;of\;WM}{\sqrt{{\left({SD}_{GM}\right)}^{2}+{({SD}_{WM})}^{2}}}$$

and the results were averaged across the four regions for comparison.

#### 2.1.3. Qualitative Image Analysis

Anonymized phantom WA datasets were presented in random order to three radiologists with varying levels of experience in neuroradiology and DECT, having sixteen, six, and three years of experience in neuroradiology, and six, four, and three years of experience in DECT, respectively. Blinded to the WF value of the reconstructed WA images, they independently evaluated noise level, GM–WM contrast, the level of posterior fossa and subcalvarial artifacts and overall image quality (IQ) using a four-point Likert scale:

1. No or minimal noise or artifacts, excellent GM/WM contrast and overall IQ.
2. Some noise and artifacts that do not influence image evaluation, very good GM/WM contrast and overall IQ.
3. Noise and artifacts that allow limited evaluation, poor GM/WM contrast and overall IQ.
4. Too much noise-uninterpretable, no GM/WM contrast, non-diagnostic images.

The analysis of axial 5 mm thick soft tissue reconstructions with a default window width set to 80 HU and the window level set at 35 HU was conducted on the same type of diagnostic monitor.

#### 2.1.4. Impact of Dose Variations on Image Quality

Considering that the CTDI_vol_ in our DECT acquisition protocol is lower than those reported by some other authors [19,36] and that delivered absorbed dose per scanning may vary across institutions and departments, it was decided to investigate the impact of a delivered larger absorbed dose on the GM–WM CNR of 0.4-WA, 0.6-WA, and 0.8-WA reconstructions. An anthropomorphic head phantom was additionally scanned with two DECT acquisition protocols in which CTDI_vol_ was increased by approximately 20% and 40% compared to the standard protocol. GM–WM CNR, SCA, and PFAI were calculated following the methodology described in Section 2.1.2.

### 2.2. Patient Study

WA reconstructions of all consecutive patients who underwent a non-contrast head DECT examination between April 2023 and July 2023 were generated. In addition to the routinely used 0.4-WA images, WA reconstructions with a WF of 0.6 and 0.8 were generated. These factors were selected based on the results of the phantom image quality analysis. The Institutional Review Board (approval number: 003-05/21-1/07) approved this prospective study and informed consent was waived due to no differences in the scanning protocol and absorbed dose to patients compared to the standard DECT protocol. Patients with brain pathologies causing significant changes in GM or WM density, shape, or volume (such as large infarcts, hemorrhages, mass lesions with mass effect, etc.) that could preclude image quality measurements and evaluation were excluded from the study. Additionally, image datasets with severe motion artifacts were also excluded. Inclusion criteria were no brain pathology or brain pathology that did not affect the specific brain regions or cross-sections that were analyzed. The DECT acquisition protocol, image processing, reconstruction parameters, and image review conditions for patient image datasets were identical to those used in the phantom study. The final patient group comprised 85 patients.

#### Quantitative and Qualitative Image Quality Analysis

**Figure 4:**
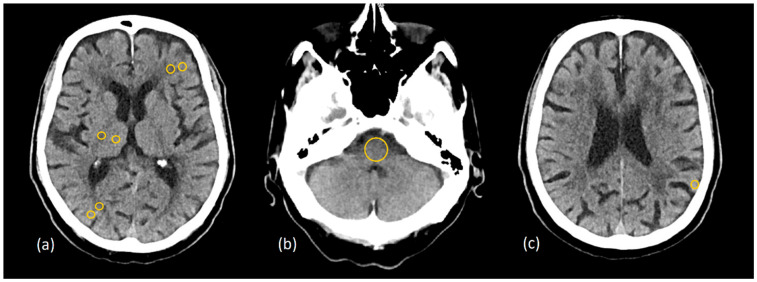
Placement of the region of interest (ROI) on the patient’s cross-sectional image data at gray matter–white matter pairs in (**a**) the frontal and parietal lobes, the thalamus, and the posterior limb of the internal capsule; (**b**) the posterior fossa; (**c**) the subcalvarial region.

Quantitative assessment of image quality followed the same methodology as described in the phantom study (Section 2.1.2). The exception in the patient study was fixed ROIs at three pairs of identical GM and WM locations in the frontal and parietal lobes and in the thalamus and adjacent posterior limb of the internal capsule, while SCA and PFAI were measured once at the level of the midbrain and near the skull, respectively (Figure 4).

**Figure 5:**
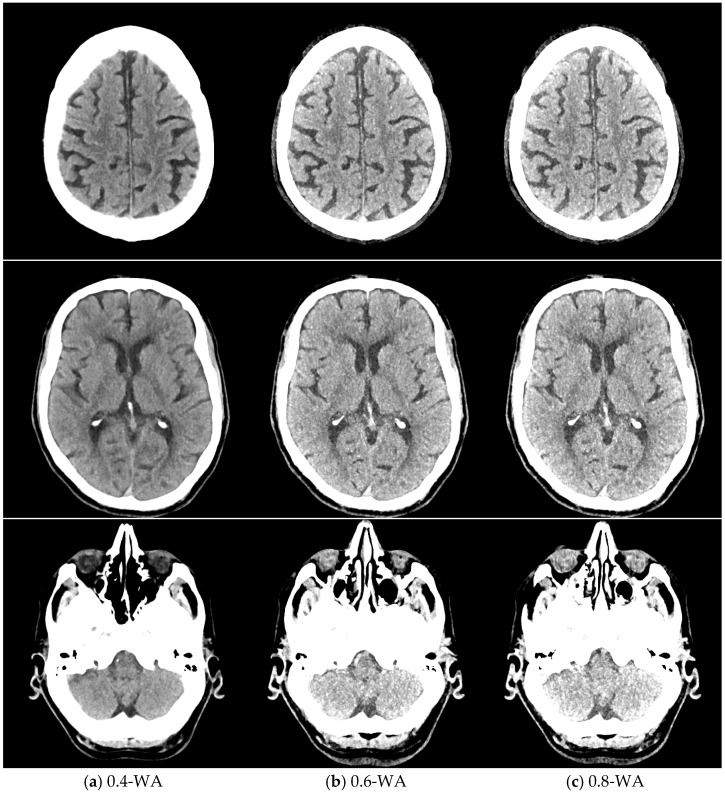
Weighted average (WA) image datasets of the brain with different weighting factors—0.4 (**a**), 0.6 (**b**), and 0.8 (**c**) of the same patient at the level of the frontoparietal lobe, basal ganglia, and posterior fossa. All images are displayed with the same window width and level (WW 80, WL 35).

The qualitative assessment of the image quality of the 0.4-WA, 0.6-WA, and 0.8-WA patient reconstructions (Figure 5) was performed according to the methodology described in Section 2.1.3. of the phantom study.

### 2.3. Statistical Analysis

Software packages MedCalc (version 22.026; MedCalc Software, Ostend, Belgium) and Statistica (version 14.1.0; TIBCO Software Inc., Palo Alto, CA, USA) were used for statistical data analysis. The significance level was set at 0.05. A power analysis was performed to determine the required sample sizes for the comparison of the different WA reconstructions. Based on the effect sizes and considering a significance level of 0.05 and a power of 0.80, a minimum sample size of 43 patients was calculated. Eventually, a total of 85 patients were included in the study, which strengthens the reliability and generalizability of our results.

For each patient, the averaged values of GM–WM CNR of three pairs of GM–WM locations were used. IQ metrics for each WA reconstruction were calculated as mean and SD. The normality distribution of the quantitative data was assessed using the Kolmogorov–Smirnov test. Normally distributed data were presented as mean ± SD and the non-parametric data as median and interquartile range (IQR). To compare normally distributed continuous variables, a repeated-measures ANOVA with Bonferroni correction was used. Ordinal data were analyzed with the Friedman signed rank test. Interrater consistency was assessed using a two-way interclass correlation coefficient (ICC) model for multiple readers [37] using the interpretation categories proposed by Koo et al. [38]. Therefore, values less than 0.5, between 0.5 and 0.75, between 0.75 and 0.9, and greater than 0.9 indicate poor, moderate, good, and excellent reliability, respectively.

## 3. Results

### 3.1. Phantom Study

The dose parameters for the non-contrast DECT brain were CTDI_vol_ = 23.2 mGy and DLP = 377.5 mGy.cm. A total of 11 WA image datasets with different WFs were reconstructed. The results of GM–WM CNR, PFAI, and SCA for different WFs are shown in Table 1.

**Figure 6:**
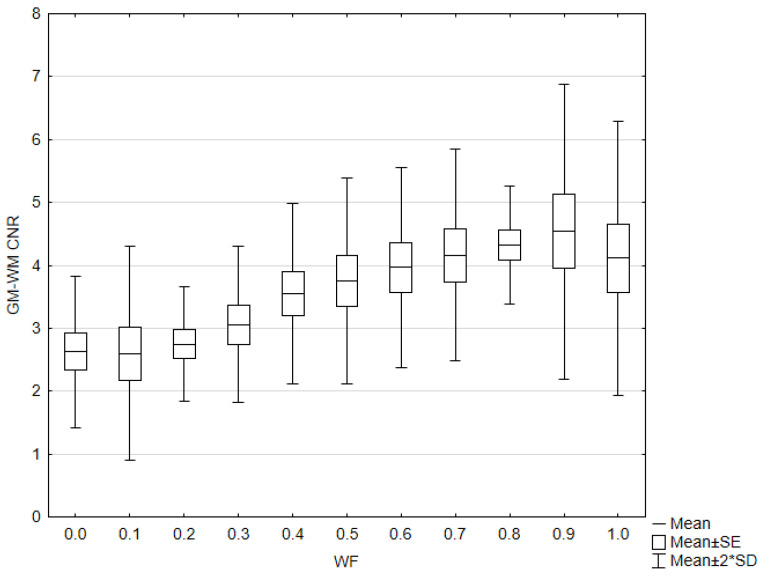
Box and whisker plot of the contrast to noise ratio (CNR) with different weighting factor (WF) in the phantom.

GM–WM CNR and noise generally increased when WF values increased from 0 to 1 (Figure 6). The 0.9-WA reconstructions had the highest CNR, and 0-WA and 0.1-WA had the lowest. SCA and PFAI were lowest for 0-WA images and highest for 1-WA images. Qualitative analysis showed unacceptable GM–WM contrast on 0-WA, 0.1-WA, and 0.2-WA image datasets for two readers. All readers rated GM–WM contrast as very good or excellent at 0.5-WA, 0.6-WA, and 0.7-WA. No or minimal posterior fossa and subcalvarial artifacts were noted at 0-WA, 0.1-WA, 0.2-WA, 0.3-WA, and 0.4-WA, while 0.9-WA and 1-WA reconstructions were considered unacceptable or for very limited evaluation by all readers. The overall IQ was rated as very good or excellent by at least two readers for the 0.5-WA, 0.6-WA, 0.7-WA, and 0.8-WA image datasets. The interrater consistency results for the assessment of the different IQ metrics for all 11 image datasets are presented in Table 2. The agreement for SCA assessment was moderate, while the interrater agreement for noise, GM–WM contrast, PFA, and overall IQ was good.

Based on the results of both quantitative and qualitative phantom analyses, WA reconstructions with WFs of 0.4, 0.6, and 0.8 were selected for the patient study. The inclusion of WF 0.4 was essential as it is routinely used in clinical practice; thus, it provides a standard for comparison. The quantitative analysis showed that the difference in GM–WM CNR between WFs 0.4 and 0.5 was smaller than between 0.5 and 0.6, and the difference between WFs 0.7 and 0.8 was also minimal. In addition, WFs lower than 0.4 and higher than 0.8 were classified as unacceptable or suitable for very limited evaluation based on the analyses and were therefore excluded from further study.

#### Impact of Dose Variations on Image Quality

The exposure and dose parameters for protocol 1 (P1), protocol 2 (P2), and protocol 3 (P3) are shown in Table 3. From each scanning protocol, 0.4-WA, 0.6-WA, and 0.8-WA image datasets were reconstructed. The results of GM–WM CNR, PFAI, and SCA are presented in Table 4.

**Figure 7:**
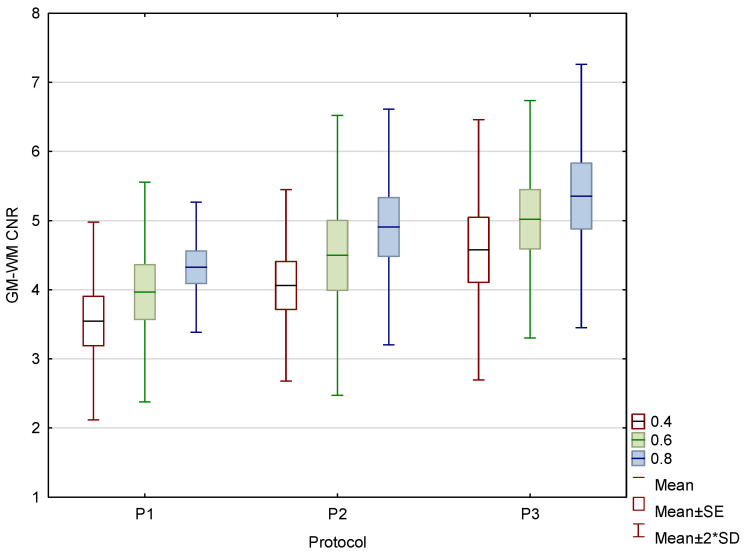
Box and whisker plot of the contrast to noise ratio (CNR) in the phantom for three protocols.

With increased CTDIvol, the GM–WM CNR improved for all three WA reconstructions (Figure 7), while noise levels decreased in the posterior fossa and near the skull.

### 3.2. Patient Study

In total, image datasets from 85 adult patients were analyzed: 35 (41%) male and 50 (59%) female, 19–90 years (median 71, IQR 15). The indications for CT scanning were headache (*n* = 35), vertigo (*n* = 23), tremor (*n* = 8), dementia (*n* = 11), transient global amnesia (*n* = 3), and syncope (*n* = 5).

The mean CTDI_vol_ was 20.8 mGy and the DLP was 347.4 mGy.cm. The mean and SD of the quantitative IQ metrics for each image dataset are presented in Table 5.

**Figure 8:**
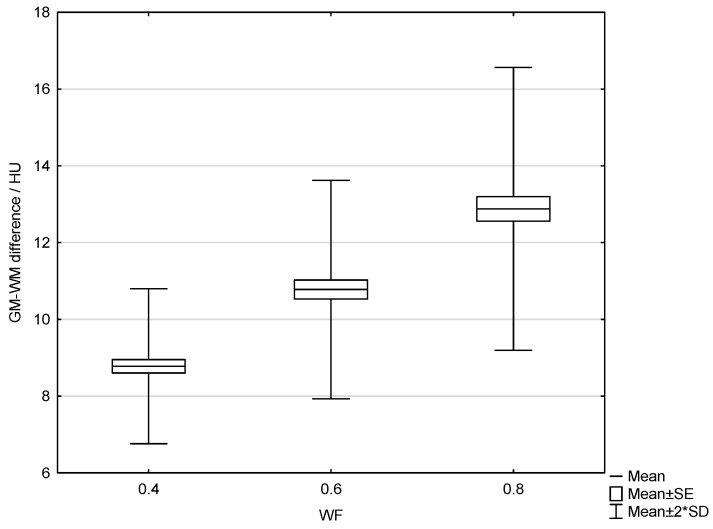
Box and whisker plot of the gray–white matter difference for 0.4, 0.6, and 0.8 weighting factor (WF) measured in patients.

**Figure 9:**
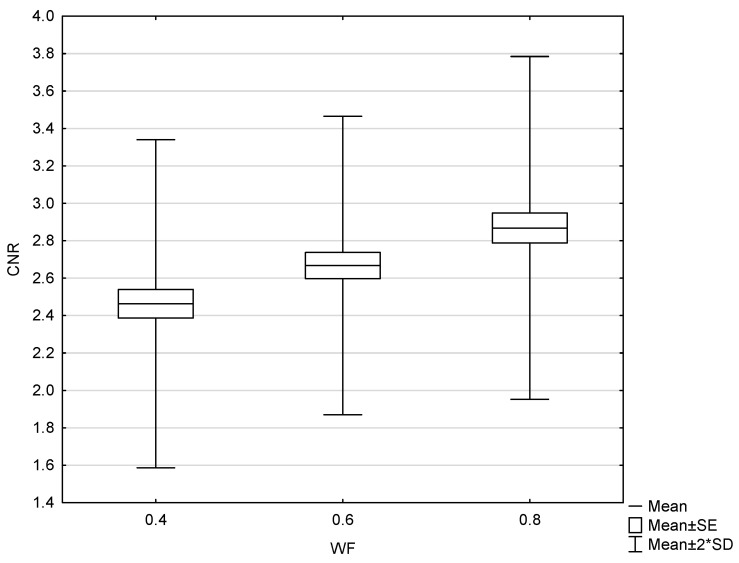
Box and whisker plot of gray–white matter contrast-to-noise radio with 0.4, 0.6, and 0.8 weighting factor (WF), measured in patients.

**Figure 10:**
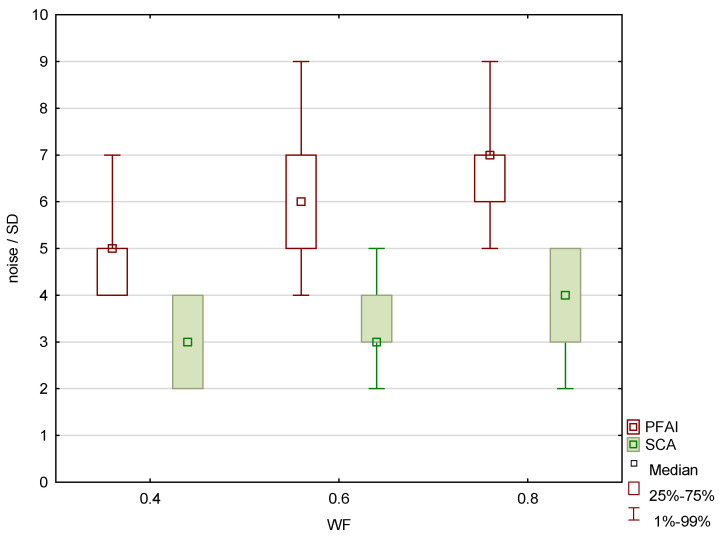
Subcalvarial artifacts (SCA) and posterior fossa artifact index (PFAI) for weighting factors 0.4, 0.6 and 0.8, measured in patients.

The results of the patient study showed the same trend as those of the phantom study. Increasing the proportion of low-energy data in 0.8-WA reconstructions compared to 0.4-WA and 0.6-WA improved GM–WM CNR and GM–WM difference (Figure 8 and Figure 9), despite simultaneously increasing noise levels (Figure 10). A repeated-measures ANOVA was performed to compare IQ metrics across three different WA image datasets, and the results showed a statistically significant difference for all IQ metrics. To control for multiple comparisons, post hoc pairwise comparisons with Bonferroni correction were performed, resulting in an adjusted significance level of *p* = 0.0167. The results showed statistically significant differences in GM–WM HU difference, GM–WM CNR, PFAI, and SCA (all *p* < 0.001) between the different WA image datasets, as presented in Table 3. The 0.8-WA GM–WM attenuation difference and CNR were significantly superior to the other WA reconstructions, while 0.4-WA had the lowest PFAI and SCA.

**Figure 11:**
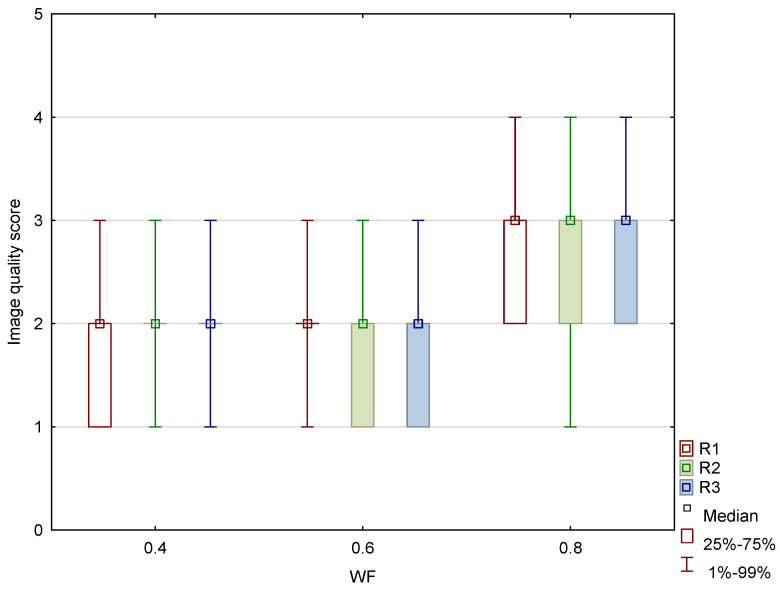
Box and whisker plot of the overall image quality score for image datasets generated with weighting factor (WF) of 0.4, 0.6, and 0.8 between three different readers (R1, R2, and R3).

The results of the qualitative analysis and image quality scores for all three readers, along with the results of the Friedman test with Bonferroni correction, are summarized in Table 6 and Figure 11. All readers found statistically significant differences in noise, SCA, and PFAI between all three WA image datasets. Reader 1’s results showed no significant differences in GM–WM contrast between the 0.4-WA and 0.6-WA images (*p* = 0.086), while reader 3 found no significant difference between the 0.4-WA and 0.8-WA reconstructions (*p* = 0.161). All three readers found that the overall IQ of 0.8-WA images was significantly lower compared to the 0.4-WA and 0.6-WA reconstructions. Two readers rated the overall IQ highest for 0.6-WA, with statistically significant differences compared to 0.4-WA and 0.8-WA (both *p* < 0.001). Reader 1 found the overall IQ of the 0.4-WA and 0.6-WA reconstructions equally superior to that of 0.8-WA (*p* < 0.001).

Interrater agreement was moderate for the assessment of the 0.4-WA GM–WM contrast, while it was good or excellent for all other qualitative IQ metrics. The detailed results are shown in Table 7.

## 4. Discussion

This study shows that the image quality of 0.6-WA image reconstructions is superior to the 0.4-WA images, which are generally recommended for the routine evaluation of non-contrast DECT of the brain. Quantitative and subjective analysis of phantom and patient images revealed that the optimal balance between GM–WM CNR, noise, and artifacts is achieved in WA reconstructions with 60% to 40% contribution of low-energy (80 kVp) and high-energy (140 kVp) datasets, respectively. While SECT provides an acceptable contrast-to-noise ratio, the 0.6-WA DECT reconstructions significantly outperformed the SECT proxy DECT reconstructions with 0.4 WF. This improved contrast between GM and WM increases the diagnostic accuracy of brain imaging, which is not as easily achieved with SECT.

As expected, GM–WM CNR was highest at high WFs, while the reconstructions with the lowest WFs exhibited minimal artifacts in the phantom scans. As anticipated, noise metrics such as SCA and PFAI decreased with lower WFs due to greater contribution of 140 kVp acquisition data to the final WA reconstructions, resulting in higher maximum X-ray beam energy and, consequently, less noise and fewer artifacts. Conversely, GM–WM CNR increased with higher WFs and a greater proportion of low-energy data in the final WA images. However, the WA reconstructions with the highest measured and subjectively perceived GM–WM CNR (WF 1 and 0.9) and those with minimal or no artifacts (WF 0, 0.1, 0.2, and 0.3) were rated overall by readers as non-diagnostic and, thus, clinically useless. The readers found that WA reconstructions with WFs of 0.5, 0.6, 0.7, and 0.8 had the best overall IQ, which indicates that the optimal balance between GM–WM contrast and artifacts is achieved at these mixed energy ratios.

By increasing CTDI_vol_ by 20% and 40% compared to the standard protocol, GM–WM CNR increased in the phantom study for each WA image dataset. Conversely, the noise metrics SCA and PFAI decreased with higher dose parameters for each WA image dataset, indicating a reduction in artifacts and overall noise. This trend is due to the larger mAs values in protocols with increased CTDI_vol_. It is known that increasing the current through the cathode (mAs) results in more photons being produced and reaching the detector, which reduces the noise in the image datasets and consequently leads to a higher CNR. The results show that the improvement in CNR between the 0.4- and 0.6-WA image datasets and between the 0.6- and 0.8-WA image datasets remained almost consistent with increasing CTDI_vol_.

Building on the phantom study, the patient study focused on 0.4-WA, 0.6-WA, and 0.8-WA image datasets. The quantitative analysis showed the same trend as in the phantom study—as expected, GM–WM CNR, SCA, and PFAI increased with higher WF. Although the absolute differences in CNR between the different WA image datasets are small, they are statistically significant and clinically relevant. Even small improvements in CNR can enhance diagnostic confidence, particularly in distinguishing subtle brain pathologies, as the inherent GM–WM contrast on CT imaging is small, with only a 5–10 HU difference in attenuation. Readers agreed that 0.6-WA reconstructions had the highest overall quality for image evaluation. We argue that a slight prevalence of low-energy data enhances GM–WM CNR while the contribution of high-energy data is still high enough to keep the artifacts at a reasonably low level. Although the routinely used 0.4-WA reconstructions, which are considered a proxy for standard SECT images at 120 kV, have fewer artifacts compared to 0.6-WA images, they are diagnostically inferior because they do not achieve sufficient GM–WM CNR, which is pivotal for detecting abnormalities in the brain tissue. On the other hand, although GM–WM CNR is higher in 0.8-WA images compared to 0.6-WA reconstructions, the artifacts affecting the analysis at the brain–bone interface (SCA) and posterior fossa (PFAI) significantly lower its overall diagnostic value. Moreover, all readers deemed 0.8-WA reconstructions to be significantly inferior to 0.4- and 0.6-WA images. These results are consistent with other studies that have shown that radiologists are more likely to prefer higher-contrast images, even at the expense of increased noise, over lower-contrast images with minimal noise artifacts [25,39].

Two readers found the 0.6-WA reconstructions superior to the 0.4-WA image data, while reader 1 found both to be equally good (*p* = 0.871). A high *p*-value indicates that the radiologist did not perceive a significant difference between these two image datasets. It is worth noting that the radiologist who found the 0.4- and 0.6-WA reconstructions to be equally good had more experience with SECT. Some studies suggest that radiologists tend to prefer imaging techniques with which they are more familiar and may exhibit biases against newer methods due to their comfort and experience with traditional techniques [40,41]. Reader 1 may have perceived the 0.4-WA images as more familiar and comfortable in terms of contrast and noise, akin to conventional SECT images commonly used in clinical practice. This familiarity may have influenced the subjective assessment and resulted in 0.4-WA images being rated with the highest score, but with no statistically significant difference compared to 0.6-WA reconstructions. In contrast, readers with more experience in DECT found 0.6-WA to be superior. This discrepancy suggests the need for training and familiarity when integrating DECT into clinical practice, which could minimize inconsistencies in future studies.

Interrater agreement was moderate only for the GM–WM contrast of the 0.4-WA image dataset. For other IQ metrics for different WFs, agreement was good or excellent. Specifically, overall IQ for the 0.4-WA and 0.6-WA showed good agreement, while the 0.8-WA exhibited excellent agreement, as all readers scored it as very poor or diagnostically unacceptable, mostly due to artifacts in the posterior fossa.

There are only a limited number of studies that have investigated the influence of different WFs on the image quality of WA reconstructions [23,25,33,34]. In accordance with our results, all authors reported that overall IQ was better in WA reconstructions with higher WF values compared to those recommended for routine clinical use. The findings of Kim et al. [25] and Behrendt et al. [33] showed the best IQ of WA reconstructions with a WF of 0.5 for post-contrast DECT of the abdomen and angiography, respectively, compared with the routinely used WF of 0.3. Tawfik et al. [23] showed that 0.6-WA reconstructions of the post-contrast DECT of the neck were superior in lesion detection to routinely used 0.3-WA reconstructions simulating standard 120 kVp SECT acquisition. The highest GM–WM CNR in the temporal lobe of post-contrast DECT of the brain in the study by Paul et al. [34] was with a WF of 0.6. Dodig et al. [28] compared the IQ of standard SECT and routinely used 0.4-WA images and concluded that the dose adjusted GM–WM CNR of 0.4-WA reconstructions was even superior to SECT images. To the best of our knowledge, no other study has evaluated the impact of different WF on the image quality of WA reconstructions that are used for routine clinical evaluation of non-contrast DECT of the brain. Given the results of our study and other cited studies, the appropriateness of using the WA DECT reconstructions that simulate SECT images for routine clinical use is questionable, as they are clearly inferior to reconstructions with different, namely, higher, WFs. Furthermore, it is counterintuitive to use DECT technology and all its post-acquisition possibilities in a way to just resemble SECT images.

There are several limitations to our study. First, to avoid confounding factors in the evaluation and measurement of IQ metrics, patients with obvious pathologies were excluded. As a result, we could not assess how changing WFs would influence lesion conspicuity and diagnostic accuracy of different WA reconstructions, which would be valuable to investigate in the future. Second, our study involved only three radiologists, all of whom had different levels of experience in neuroradiology. This limited number of readers may not fully capture the variability in image assessment that could arise with a more diverse group of radiologists, especially those with less experience in neuroradiology. However, by including a resident, a younger specialist, and a senior specialist in neuroradiology, we aimed to reflect a realistic range of experience in clinical practice within a medium-sized radiology department and provide a balanced representation of the perspectives involved in clinical image evaluation. It is important to note that the inclusion of a wider pool of readers, especially those with more varying levels of expertise, could offer a more comprehensive understanding of image quality assessment. Finally, CTDI_vol_ in our DECT protocol is much lower than in some other studies [19,36]. Tijssen et al. [19] reported a CTDI_vol_ of 37 mGy and Zhao et al. [36] of 30.19 mGy, while the typical value of the dose indicator CTDI_vol_ in the standard non-contrast head DECT protocol in our hospital is 21.8 mGy and could be considered almost low-dose protocol. We argue that the DECT brain protocol in our hospital could be further optimized by increasing the dose delivered to the patient.

Although Dodig [28] showed comparable IQ in SECT and DECT brain images on the same scanner in our hospital, we believe that our DECT brain protocol should be further refined by increasing the reference mAs and, consequently, the CTDI_vol_. The results from the IQ assessment in a phantom with different dose parameters indicated that GM–WM CNR increased with higher CTDI_vol_. The analysis was conducted for only three protocols with different dose parameters and three WA image datasets, as this was not the primary focus of this study. A more comprehensive analysis, incorporating additional protocols with varying CTDI_vol_, will be conducted in the future.

Our findings are only applicable to non-contrast DECT of the brain and CT indications that do not involve the use of contrast agents, as this study focused only on the technical evaluation of image quality and not on the assessment of diagnostic accuracy. However, we are confident that our results on the impact of different WFs on image quality could serve as a prerequisite for future clinical studies elsewhere. Such studies should explore the influence of different WFs on the diagnostic accuracy of both non-contrast and post-contrast DECT scans of the brain, complementing and expanding upon our results.

## 5. Conclusions

This study shows that modifying the WF for non-contrast WA DECT reconstructions of the brain significantly affects IQ metrics. DE 0.6-WA images exhibit superior GM–WM CNR and overall image quality compared to the routinely used 0.4-WA reconstructions. Therefore, we recommend the use of a 0.6-WA reconstruction instead of the 0.4-WA reconstructions for routine evaluation of non-contrast DECT brain images.

## Figures and Tables

**Table 1:** Quantitative image quality metrics of the phantom study for weighted average image datasets with different weighting factor (WF) expressed as mean (SD).

WF	IQ Metric		
	GM–WM CNR	PFAI	SCA
0	2.6 (0.6)	4.8 (0.2)	2.1 (0.5)
0.1	2.6 (0.8)	5.6 (0.4)	2.3 (0.4)
0.2	2.7 (0.5)	6.7 (0.1)	2.5 (0.3)
0.3	3.1 (1.6)	6.8 (0.2)	2.7 (0.4)
0.4	3.5 (0.7)	7.5 (0.2)	2.9 (0.5)
0.5	3.7 (0.8)	7.8 (0.2)	3.0 (0.7)
0.6	4.0 (0.8)	8.7 (0.1)	3.3 (0.7)
0.7	4.2 (0.8)	8.7 (0.4)	3.6 (0.9)
0.8	4.3 (0.5)	9.6 (0.4)	3.7 (0.9)
0.9	4.6 (1.1)	9.9 (0.3)	3.8 (0.8)
1	4.1 (1.1)	10.7 (0.3)	3.9 (0.9)

WF: weighting factor; GM: gray matter; WM: white matter; CNR: contrast-to-noise ratio; PFAI: posterior fossa artifact index; SCA: subcalvarial artifacts.

**Table 2:** Interrater consistency results for the assessment of different IQ metrics across all eleven image datasets presented with interclass correlation coefficient (ICC) and confidence interval (CI).

IQ Metric	ICC	95% CI
Noise	0.87	0.65–0.96
GM/WM contrast	0.86	0.61–0.96
SCA	0.71	0.19–0.92
PFAI	0.86	0.63–0.96
Overall IQ	0.81	0.49–0.94

GM: gray matter; WM: white matter; SCA: subcalvarial artifacts; PFAI: posterior fossa artifact index.

**Table 3:** Exposure and dose parameters for P1, P2, and P3.

Protocol	kVp	Quality Reference mAs	CTDIvol/mGy	DLP/mGy.cm
P1	80/140 Sn	310 tube A (80 kV)	23.2	377.5
		155 tube B (Sn 140 kV)		
P2	80/140 Sn	373 tube A (80 kV)	27.5	447.4
		187 tube B (Sn 140 kV)		
P3	80/140 Sn	445 tube A (80 kV)	33.0	520.6
		223 tube B (Sn 140 kV)		

P1: standard DECT protocol, P2: standard DECT protocol with 20% higher dose parameters, P3: standard DECT protocol with 40% higher dose parameters.

**Table 4:** Quantitative image quality metrics of the phantom study for 0.4-, 0.6-, and 0.8-weighted average image datasets for different dose parameters expressed as mean (SD).

CTDI_vol_/mGy	WF	IQ Metric		
		GM–WM CNR	PFAI	SCA
23.2	0.4	3.5 (0.7)	7.5 (0.2)	2.9 (0.5)
	0.6	4.0 (0.8)	8.7 (0.1)	3.5 (1.2)
	0.8	4.3 (0.5)	9.6 (0.4)	3.7 (0.9)
27.5	0.4	4.1 (0.7)	7.1 (0.1)	2.5 (0.1)
	0.6	4.5 (1.0)	8.0 (0.2)	2.7 (0.4)
	0.8	4.9 (0.9)	9.2 (0.2)	3.2 (0.5)
33.0	0.4	4.6 (0.9)	6.9 (0.1)	2.0 (0.3)
	0.6	5.0 (0.9)	7.9 (0.1)	2.4 (0.5)
	0.8	5.4 (1.0)	9.0 (0.2)	2.6 (0.6)

GM: gray matter; WM: white matter; CNR: contrast-to-noise ratio; PFAI: posterior fossa artifact index; SCA: subcalvarial artifacts.

**Table 5:** Results of quantitative weighted average (WA) image quality (IQ) metrics in the patient study for different weighting factor (WF). Gray matter (GM) to white matter (WM) attenuation difference and GM–WM contrast-to-noise ratio (CNR) are expressed as mean (SD), and subcalvarial artifacts (SCA) and posterior fossa artifact index (PFAI) as median (IQR).

IQ Metric	WA Image Dataset					
	WF 0.4	WF 0.6	WF 0.8	*p*-Value (0.4 vs. 0.6)	*p*-Value (0.6 vs. 0.8)	*p*-Value (0.4 vs. 0.8)
GM–WM HU difference	8.8 (1.0)	10.8 (1.4)	12.9 (1.8)	<0.001	<0.001	<0.001
GM–WM CNR	2.5 (0.4)	2.7 (0.4)	2.9 (0.5)	<0.001	<0.001	<0.001
SCA	3 (2)	3 (1)	4 (2)	<0.001	<0.001	<0.001
PFAI	5 (1)	6 (2)	7 (1)	<0.001	<0.001	<0.001

**Table 6:** Results of qualitative image quality (IQ) assessment of three readers for weighted average image datasets reconstructed with weighted factor (WF) 0.4, 0.6, and 0.8 presented as median (IQR).

		WA Image Dataset					
**IQ Metric**	**Reader**	**WF 0.4**	**WF 0.6**	**WF 0.8**	***p*-value** **(0.4 vs. 0.6)**	***p*-value** **(0.6 vs. 0.8)**	***p*-value** **(0.4 vs. 0.8)**
	R 1	1 (0)	1 (1)	2 (1)	<0.001	<0.001	<0.001
Noise	R 2	1 (0)	1 (1)	2 (1)	<0.001	<0.001	<0.001
	R 3	1 (0)	1 (1)	2 (1)	<0.001	<0.001	<0.001
	**Reader**	**WF 0.4**	**WF 0.6**	**WF 0.8**	***p*-value** **(0.4 vs. 0.6)**	***p*-value** **(0.6 vs. 0.8)**	***p*-value** **(0.4 vs. 0.8)**
	R 1	1 (1)	2 (1)	2 (1)	0.086 *	<0.001	<0.001
GM/WM contrast	R 2	2 (0)	2 (1)	2 (0)	<0.001	<0.001	<0.001
	R 3	2 (0)	2 (1)	2 (0)	<0.001	<0.001	0.161 *
	**Reader**	**WF 0.4**	**WF 0.6**	**WF 0.8**	***p*-value** **(0.4 vs. 0.6)**	***p*-value** **(0.6 vs. 0.8)**	***p*-value** **(0.4 vs. 0.8)**
	R 1	1 (1)	2 (1)	3 (1)	<0.001	<0.001	<0.001
SCA	R 2	1 (0)	1 (1)	2 (1)	<0.001	<0.001	<0.001
	R 3	1 (0)	1 (1)	2 (1)	<0.001	<0.001	<0.001
	**Reader**	**WF 0.4**	**WF 0.6**	**WF 0.8**	***p*-value** **(0.4 vs. 0.6)**	***p*-value** **(0.6 vs. 0.8)**	***p*-value** **(0.4 vs. 0.8)**
	R 1	2 (0)	2 (1)	3 (0)	<0.001	<0.001	<0.001
PFAI	R 2	2 (0)	2 (1)	3 (0)	0.001	<0.001	<0.001
	R 3	2 (0)	2 (1)	3 (0)	0.005	<0.001	<0.001
	**Reader**	**WF 0.4**	**WF 0.6**	**WF 0.8**	***p*-value** **(0.4 vs. 0.6)**	***p*-value** **(0.6 vs. 0.8)**	***p*-value** **(0.4 vs. 0.8)**
	R 1	2 (0)	2 (1)	3 (1)	0.871 *	<0.001	<0.001
Overall IQ	R 2	2 (0)	2 (1)	3 (1)	<0.001	<0.001	<0.001
	R 3	2 (0)	2 (1)	3 (1)	0.013	<0.001	<0.001

GM: gray matter; WM: white matter; SCA: subcalvarial artifacts; PFAI: posterior fossa artifact index; * statistically not significant.

**Table 7:** Interrater agreement results of the qualitative assessment of different weighted average patient datasets at weighted factor (WF) 0.4, 0.6, and 0.8.

IQ Metric	WA Image Dataset	ICC	95% CI
Noise	0.4	0.88	0.83–0.92
	0.6	0.95	0.93–0.97
	0.8	0.95	0.92–0.97
GM/WM contrast	0.4	0.65	0.20–0.83
	0.6	0.83	0.76–0.89
	0.8	0.76	0.52–0.87
SCA	0.4	0.82	0.74–0.88
	0.6	0.91	0.88–0.94
	0.8	0.96	0.94–0.97
PFAI	0.4	0.87	0.82–0.91
	0.6	0.85	0.78–0.90
	0.8	0.91	0.87–0.94
Overall IQ	0.4	0.86	0.80–0.91
	0.6	0.86	0.80–0.90
	0.8	0.95	0.94–0.97

IQ: image quality; ICC: interrater correlation coefficient; CI: confidence interval; GM: gray matter; WM: white matter; SCA: subcalvarial artifacts; PFAI: posterior fossa artifact index.

## Data Availability

All data generated or analyzed during this study are included in this article. Further enquiries can be directed to the corresponding author.
